# Targeting Cytokine Release Through the Differential Modulation of Nrf2 and NF-κB Pathways by Electrophilic/Non-Electrophilic Compounds

**DOI:** 10.3389/fphar.2020.01256

**Published:** 2020-08-14

**Authors:** Francesca Fagiani, Michele Catanzaro, Erica Buoso, Filippo Basagni, Daniele Di Marino, Stefano Raniolo, Marialaura Amadio, Eric H. Frost, Emanuela Corsini, Marco Racchi, Tamas Fulop, Stefano Govoni, Michela Rosini, Cristina Lanni

**Affiliations:** ^1^Department of Drug Sciences, Pharmacology Section, University of Pavia, Pavia, Italy; ^2^Scuola Universitaria Superiore IUSS Pavia, Pavia, Italy; ^3^Department of Pharmacy and Biotechnology, University of Bologna, Bologna, Italy; ^4^Department of Life and Environmental Sciences, New York-Marche Structural Biology Center (NY-MaSBiC), Polytechnic University of Marche, Ancona, Italy; ^5^Università della Svizzera Italiana (USI), Faculty of Biomedical Sciences, Institute of Computational Science—Center for Computational Medicine in Cardiology, CH-Lugano, Switzerland; ^6^Department of Microbiology and Infectiology, Centre de Recherches Cliniques, Faculty of Medicine and Health Sciences, University of Sherbrooke, Sherbrooke, QC, Canada; ^7^Department of Environmental Science and Policy, Università degli Studi di Milano, Milan, Italy; ^8^Geriatric Division, Department of Medicine, Faculty of Medicine and Health Sciences, Research Center on Aging, University of Sherbrooke, Sherbrooke, QC, Canada

**Keywords:** Nrf2, NF-κB, curcumin, antioxidant, inflammation, cytokine release, TNFα, MCP-1

## Abstract

The transcription factor Nrf2 coordinates a multifaceted response to various forms of stress and to inflammatory processes, maintaining a homeostatic intracellular environment. Nrf2 anti-inflammatory activity has been related to the crosstalk with the transcription factor NF-κB, a pivotal mediator of inflammatory responses and of multiple aspects of innate and adaptative immune functions. However, the underlying molecular basis has not been completely clarified. By combining into new chemical entities, the hydroxycinnamoyl motif from curcumin and the allyl mercaptan moiety of garlic organosulfur compounds, we tested a set of molecules, carrying (pro)electrophilic features responsible for the activation of the Nrf2 pathway, as valuable pharmacologic tools to dissect the mechanistic connection between Nrf2 and NF-κB. We investigated whether the activation of the Nrf2 pathway by (pro)electrophilic compounds may interfere with the secretion of pro-inflammatory cytokines, during immune stimulation, in a human immortalized monocyte-like cell line (THP-1). The capability of compounds to affect the NF-κB pathway was also evaluated. We assessed the compounds-mediated regulation of cytokine and chemokine release by using Luminex X-MAP^®^ technology in human primary peripheral blood mononuclear cells (PBMCs) upon LPS stimulation. We found that all compounds, also in the absence of electrophilic moieties, significantly suppressed the LPS-evoked secretion of pro-inflammatory cytokines such as TNFα and IL-1β, but not of IL-8, in THP-1 cells. A reduction in the release of pro-inflammatory mediators similar to that induced by the compounds was also observed after siRNA mediated-Nrf2 knockdown, thus indicating that the attenuation of cytokine secretion cannot be directly ascribed to the activation of Nrf2 signaling pathway. Moreover, all compounds, with the exception of compound 1, attenuated the LPS-induced activation of the NF-κB pathway, by reducing the upstream phosphorylation of IκB, the NF-κB nuclear translocation, as well as the activation of NF-κB promoter. In human PBMCs, compound 4 and CURC attenuated TNFα release as observed in THP-1 cells, and all compounds acting as Nrf2 inducers significantly decreased the levels of MCP-1/CCL2, as well as the release of the pro-inflammatory cytokine IL-12. Altogether, the compounds induced a differential modulation of innate immune cytokine release, by differently regulating Nrf2 and NF-κB intracellular signaling pathways.

## Introduction

Nuclear factor (erythroid-derived 2)-like 2 (Nrf2) is a transcription factor regulating the expression of about 250 genes encoding a network of cooperating enzymes involved in endobiotic and xenobiotic biotransformation reactions, antioxidant metabolism, protein degradation and regulation of inflammation (Hayes and Dinkova-Kostova, 2014). By governing such complex transcriptional networks, Nrf2 coordinates a multifaceted response to various forms of stress, maintaining a homeostatic intracellular environment. Several studies demonstrate that Nrf2 plays also a key role in the resolution of inflammatory processes. Consistently, Nrf2 is abundant in monocytes and granulocytes, proving its crucial involvement in immune response driven by these cell types. Data from the literature demonstrate that genetic or pharmacological activation of Nrf2 strongly suppresses the production of pro-inflammatory cytokines (Innamorato et al., 2008; Knatko et al., 2015; Kobayashi et al., 2016; Quinti et al., 2017) and Nrf2-deficiency induces an exacerbation of inflammation in a variety of murine models such as sepsis, pleurisy, and emphysema (Iizuka et al., 2005; Ishii et al., 2005; Thimmulappa et al., 2006). However, while the contribution of Nrf2 in inflammatory processes has been widely recognized, the underlying molecular basis has not been completely clarified. Its anti-inflammatory activity has been related to several mechanisms, including crosstalk with the transcription factor nuclear factor-κB (NF-κB), the modulation of redox balance, and the direct down-regulation of some antioxidant response element (ARE)-dependent expression of pro-inflammatory cytokines, such as IL-6 and IL-1β (Kobayashi et al., 2016). Among them, the crosstalk between Nrf2 and NF-κB relies on both transcriptional and post-transcriptional mechanisms, allowing fine-tuning of dynamic responses to ever-changing environmental cues. NF-κB is a key transcription factor governing the expression of a plethora of genes involved in diverse biological processes, including immune and inflammatory responses, cell proliferation, death, angiogenesis, cell survival, and oncogenesis (Häcker and Karin, 2006; Perkins, 2007). In particular, NF-κB controls the transcription of genes encoding pro-inflammatory cytokines, such as TNFα and IL-1β. In the absence of a stimulant, NF-κB remains inactive and sequestrated in the cytoplasm by binding to an inhibitory protein, IκB. The exposure of cells to pro-inflammatory stimuli, such as cytokines and infectious agents, triggers the activation of the IκB kinase (IKK) complex that phosphorylates IκB protein on two serine residues. Phosphorylated IκB is ubiquitinated and, subsequently, degraded *via* proteasome (Hayden et al., 2006; Perkins, 2007). The degradation of IκB allows NF-κB translocation into the nucleus to drive the expression of target genes; within the nucleus it interacts with other transcription factors and transcriptional co-factors to regulate expression of an array of genes, many of which are involved in inflammatory signaling (e.g. cytokines, chemokines, adhesion molecules, and acute phase proteins) (Baeuerle and Baltimore, 1996). Notably, several pharmacological and genetic studies suggest a functional crosstalk between Nrf2 and NF-κB transcription factors, with a range of complex molecular interactions depending on the cell type and tissue context (Wardyn et al., 2015). A strong activity in both NF-κB and Nrf2 has been found fundamental for well-coordinated responses to counteract a cellular inflammatory status (Fusco et al., 2017; D’amico et al., 2019). Indeed, an imbalance between Nrf2 and NF-κB pathways has been associated with a variety of diseases ranging from neurodegeneration, cardiovascular and autoimmune disorders.

The transcriptional factor Nrf2, with its redox sensitive repressor Keap1 (Kelch-like ECH-associated protein 1), orchestrates adaptive responses to diverse forms of stress through regulatory cysteine switches. Thus, precise electrophilic addition is emerging as a valuable opportunity to shed light on previously untapped roles of this redox sensing system (Basagni et al., 2019). By combining into new chemical entities the hydroxycinnamoyl motif derived from curcumin and the allyl mercaptan moiety of garlic organosulfur compounds, we previously synthesized a set of molecules (compounds 1-3), carrying, with the exception of compound 3, a catechol moiety and/or an α,β-unsaturated carbonyl group (**Table 1**). These (pro)electrophilic features were shown to be responsible for the activation of the Nrf2 pathway and the subsequent induction of ARE-dependent target genes, possibly by covalent conjugation with Keap1 cysteine sensors (Simoni et al., 2017; Serafini et al., 2019). Notably, alkylation of functionally significant cysteines of NF-κB was also shown to play a prominent role in the inhibition of pro-inflammatory transcriptional pathways (Kastrati et al., 2016), albeit alternative mechanisms have been proposed, such as the inhibition of IKKβ, or promotion of RelA polyubiquitination and proteasomal degradation (Woodcock et al., 2018).

**Table 1:** Design strategy of electrophilic and non-electrophilic compounds.

Reference compound			
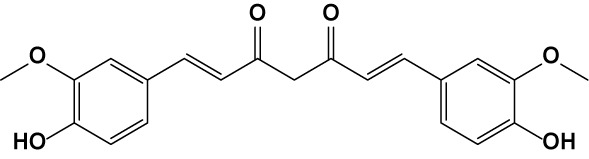			
**Curcumin**			
**Electrophilic**	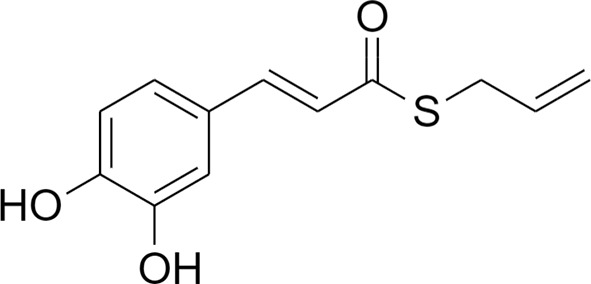 **1**	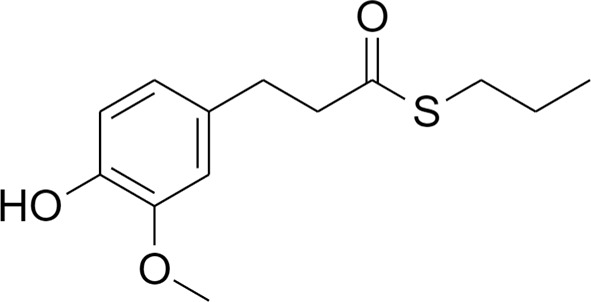 **3**	**Non-Electrophilic**
	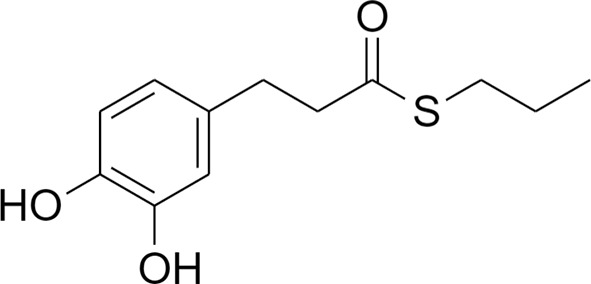 **2**	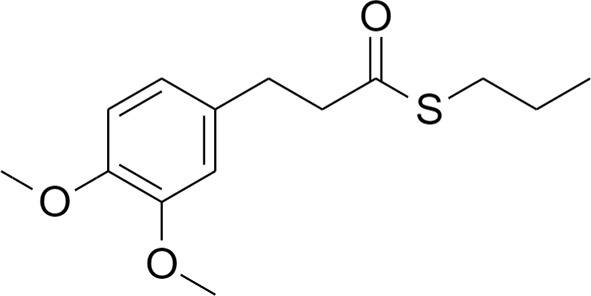 **4**	
**Originated compounds**			

Compound 1: S-allyl (E)-3-(3,4-dihydroxyphenyl)prop-2-enethioate;

Compound 2: S-propyl 3-(3,4-dihydroxyphenyl)propanethioate;

Compound 3: S-propyl 3-(4-hydroxy-3-methoxyphenyl)propanethioate;

Compound 4: S-propyl 3-(3,4-dimethoxyphenyl)propanethioate.

Herein, we considered the abovementioned compounds as valuable pharmacologic tools to explore the mechanistic connection between Nrf2 and NF-κB. To exclude possible oxidative activation of the methoxyphenol ring of compound 3 into reactive metabolites such as quinone methide, which could provide an additional electrophilic site (Luis et al., 2018), an additional new compound 4 was synthesized (**Table 1**).

In the present work, we investigated whether the modulation in Nrf2 pathway activation by our molecules was able to interfere with the LPS-induced secretion of pro-inflammatory cytokines, during immune stimulation, in a human immortalized monocyte-like cell line (THP-1), a well-established cell model for the immune modulation approach (Chanput et al., 2014), using curcumin (CURC) as a reference compound. Moreover, the capability of compounds to affect the NF-κB intracellular pathway, a pivotal mediator of inflammatory responses and critical regulator of multiple aspects of both innate and adaptative immune functions, was also investigated. To validate the results obtained in THP-1 cells in a human primary cellular model, we assessed the regulation of cytokine and chemokine (e.g. IFNγ, IL-1β, IL-4, IL-6, IL-8, IL-12 (p40), IL-12 (p70), IL-13, IL-27, MCP-1, MCP-3, TNFα) release by the described compounds, upon immune LPS stimulation, in human peripheral blood mononuclear cells (PBMCs), obtained from venous whole blood of healthy patients, by using Luminex X-MAP^®^ technology.

Altogether, we demonstrated that compounds modulated the innate immune cytokine release, by differently regulating Nrf2 and NF-κB intracellular signaling pathways.

## Materials and Methods

### Compounds Synthesis

Compounds 1–3 were synthesized according to procedures reported in (Simoni et al., 2016; Simoni et al., 2017); details on the newly synthesized compound 4 are reported here below.

#### Synthesis of S-Propyl 3-(3,4-Dimethoxyphenyl)propanethioate (Compound 4)

To a solution of compound 2 (110 mg, 0.46 mmol) in 1.80 mL of DMF potassium carbonate (222.5 mg, 1.61 mmol) and methyl iodide dropwise (0.10 mL, 1.61 mmol) were added. The reaction mixture was left stirring at room temperature overnight. The reaction was quenched by adding 3 mL of water and the mixture obtained was further extracted with diethyl ether (2 x 5 mL). Organic phases were collected, reunited, dried with anhydrous sodium sulphate and solvent was evaporated under vacuum. The crude oil was purified by column chromatography on silica gel using petroleum ether/ethyl acetate (8/2) as mobile phase. 4 was obtained as colorless oil (110 mg, 89%). 1H NMR (400 MHz, CDCl3) δ 6.74 (d, J = 8 Hz, 1H), 6.69–6.67 (m, 2H), 3.82 (s, 3H), 3.80 (s, 3H), 2.89 (t, J = 8 Hz, 2H), 2.83–2.77 (m, 4H), 1.59-1.50 (m, 2H), 0.91 (t, J = 8 Hz, 3H). 13C NMR (100 MHz, CDCl3) δ 198.79, 148.98, 147.61, 132.83, 120.27, 111.77, 111.41, 55.98, 55.89, 45.88, 31.23, 30.87, 23.06, 13.39. MS [ESI+] m/z 291.10 [M+Na]^+^.

Chromatographic separations were performed on silica gel columns (Kieselgel 40, 0.040–0.063 mm, Merck). Reactions were followed by TLC on Merck (0.25 mm) glass-packed precoated silica gel plates (60 F254), then visualized with a UV lamp. NMR spectra were recorded at 400 MHz for 1H and 100 MHz for 13C on a Varian VXR 400 spectrometer (**Supplementary Figure 1**). Chemical shifts (δ) are reported in parts per million (ppm) relative to tetramethylsilane (TMS), and spin multiplicities are given as s (singlet), br s (broad singlet), d (doublet), t (triplet), q (quartet), or m (multiplet). Direct infusion ESI-MS mass spectra were recorded on a Waters ZQ 4000 and Xevo G2-XS QTof apparatus. Final compounds were >95% pure as determined by High Performance Liquid Chromatography (HPLC) analyses. The analyses were performed under reversed-phase conditions on a Phenomenex Jupiter C18 (150 × 4.6 mm I.D.) column, using a binary mixture of H_2_O/acetonitrile (60/40, v/v for 1; 50/50, v/v for 2 and 3; 40/60, v/v for 4) as the mobile phase, UV detection at λ= 302 nm (for 1) or 254 nm (for 2–4), and a flow rate of 0.7 mL/min. Analyses were performed on a liquid chromatograph model PU-1587 UV equipped with a 20 µL loop valve (Jasco Europe, Italy).

All compounds were solubilized in DMSO (dimethyl sulfoxide) at stock concentrations of 10 mM, frozen (−20°C) in aliquots and diluted in culture medium immediately prior to use. For each experimental setting, a stock aliquot was thawed and diluted to minimize repeated freeze and thaw damage. The final concentration of DMSO in culture medium was less than 0.1%.

### Reagents

CURC (#08511) was ≥98% pure (HPLC) and purchased by Sigma-Aldrich (Merck KGaA, Darmstadt, Germany). Cell culture media and all supplements were purchased from Sigma Aldrich (Merck KGaA, Darmstadt, Germany). Rabbit polyclonal anti-human Nrf2 (NBP1-32822) and anti-human HO-1 (NBP1-31341) antibodies were purchased from Novus (Biotechne, Minneapolis USA). Mouse monoclonal anti-human IκBα (#4814T), mouse monoclonal anti-human phospho-IκBα (Ser32/36) (#9246T), and rabbit monoclonal anti-human NF-κB p65 (D14E12) XP^®^ (#8242) were purchased from Cell Signaling (Cell Signaling Technology, Danvers, MA, USA). Mouse monoclonal anti-human lamin A/C (612162) antibody was purchased from BD Biosciences (Franklin Lakes, NJ, USA). Mouse anti-human α-tubulin (sc-5286) was purchased from Sigma-Aldrich (Merck KGaA, Darmstadt, Germany). Peroxidase conjugate-goat anti-mouse (A4416) was purchased from Sigma-Aldrich (Merck KGaA, Darmstadt, Germany). Anti-rabbit peroxidase-linked antibody (#7074) was purchased from Cell Signaling (Cell Signaling Technology, Danvers, MA, USA). Lipopolysaccharide (LPS) from *Escherichia coli* O111:B4 (L2630) was purchased from Sigma-Aldrich (Merck KGaA, Darmstadt, Germany). The proteasome inhibitor MG132 (474790) was purchased from Calbiochem (San Diego, CA).

### Cell Culture and Treatments

Human THP-1 cells were purchased from the European Collection of Authenticated Cell Cultures (ECACC, Salisbury, UK) and diluted to 10^6^ cells/mL in RPMI 1640 medium supplemented with 10% heat-inactivated Fetal Bovine Serum (FBS), 2 mM glutamine, 0.1 mg/mL streptomycin, 100 IU·mL penicillin, and 0.05 mM 2-mercaptoethanol (complete medium) and maintained at 37°C in 5% CO_2_-containing and 95% air atmosphere. The experiments were carried out on passages 5–15. Cells were treated as reported in figure legends. Control cells were exposed only to solvent (DMSO).

### Cell Viability

The mitochondrial dehydrogenase activity that reduces 3-(4,5-dimethylthiazol-2-yl)-2,5-diphenyl-tetrazolium bromide (MTT, Sigma Aldrich, Merck KGaA, Darmstadt, Germany) was used to determine cell viability using a quantitative colorimetric assay (Kumar et al., 2018). At day 0, THP-1 cells were plated in 96-well plates at a density of 50 x 10^3^ viable cells per well. After treatment, according to the experimental setting, cells were exposed to an MTT solution (1 mg/mL) in complete medium. After 4 h of incubation with MTT, cells were lysed with sodium dodecyl sulfate (SDS) for 24 h and cell viability was quantified by reading absorbance at 570 nm wavelength, using Synergy HT multi-detection microplate reader (Bio-Tek, Winooski, VT, USA).

### Subcellular Fractionation for Nrf2 and NF-κB Nuclear Translocation

The expression of Nrf2 and NF-κB in nuclear THP-1 lysates was assessed by Western blot analysis. Suspended cells were collected, centrifugated, and washed twice with ice-cold PBS (phosphate buffered saline), and, subsequently, homogenized 20 times using a glass-glass homogenizer in ice-cold fractionation buffer (20 mM Tris/HCl pH 7.4, 2 mM EDTA, 0.5 mM EGTA, 0.32 M sucrose, 50 mM β-mercaptoethanol). The homogenate was centrifuged at 300 × *g* for 5 min to obtain the nuclear fraction. An aliquot of the nuclear extract was used for protein quantification by the Bradford method, whereas the remaining sample was boiled at 95°C for 5 min after dilution with 2X sample buffer (125 mM Tris-HCl pH 6.8, 4% SDS, 20% glycerol, 6% β-mercaptoethanol, 0.1% bromophenol blue). Equivalent amounts of nuclear extracted proteins (30 μg) were subjected to polyacrylamide gel electrophoresis and immunoblotting, as described below.

### Immunodetection of Nrf2, HO-1, p-IκBα, IκBα, and NF-κB

The expression of Nrf2, HO-1, p-IκBα, IκBα, and NF-κB in whole cell lysates or nuclear extracts was assessed by Western blot analysis. After treatments, suspended cells were collected, centrifugated, and washed twice with ice-cold PBS, lysed by the addition of ice-cold homogenization buffer (50 mM Tris-HCl pH 7.5, 150 mM NaCl, 5 mM EDTA, 0.5% Triton X-100 and protease-phosphatase inhibitors mix). Samples were sonicated and centrifuged at 13,000 x *g* for 10 s at 4°C. The resulting supernatants were transferred into new tubes, and protein content was determined by Bradford method. After that, the samples were boiled at 95°C for 5 min after dilution with 5X sample buffer. For Western blot analysis, equivalent amounts of both total and nuclear extracts (30 μg) were electrophoresed in 10% acrylamide gel, under reducing conditions, then, electroblotted into PVDF membranes (Sigma Aldrich, Merck KGaA, Darmstadt, Germany), blocked for 1 h with 5% w/v bovine serum albumin (BSA) in TBS-T (0.1 M Tris-HCl pH 7.4, 0.15 M NaCl, and 0.1% Tween 20), and incubated overnight at 4°C with primary antibodies diluted in 5% w/v BSA in TBS-T. The proteins were visualized using primary antibodies for Nrf2 (1:1000), HO-1 (1:1000), IκBα (1:1000), p-IκBα (1:1000), or NF-κB (1:1000). Detection was carried out by incubation with secondary horseradish peroxidase-conjugated antibodies (1:5000) diluted in 5% w/v BSA in TBS-T for 1 h at room temperature. Membranes were subsequently washed three times with TBS-T and proteins of interest were visualized using an enhanced chemiluminescent reagent (Pierce, Rockford, IL, USA). α-tubulin and lamin A/C were performed as controls for gel loading.

### Small Interference RNA (siRNA) for Nrf2

Nrf2 siRNA designed for the human gene *Nrf2* was purchased from Sigma Aldrich, Merck KGaA (Darmstadt, Germany). A scrambled siRNA, without known homology with any gene, was used as negative control (Sigma Aldrich, Merck KGaA, Darmstadt, Germany). RNA interference experiments in THP-1 cells were performed by transient transfection for 24 h, using RNAiMAX Lipofectamine (Invitrogen, Thermo Fisher Scientific, Waltham, MA, USA), according to manufacturer’s protocol. To confirm Nrf2 silencing, the proteasome inhibitor MG132 (Calbiochem) was added to the medium of selected plates at a final concentration of 5 µM. After 24 h, cells were analyzed for Nrf2 expression by Western blot analysis.

### Enzyme-Linked Immunosorbent Assay (ELISA) Determination of TNFα, IL-8, and IL-1β

THP-1 cells were treated with compounds 1–4 and CURC at a concentration of 5 μM for 24 h, and then stimulated with LPS for 3 h, as described in the legends to figures. TNFα, IL-8, and IL-1β released from THP-1 cells were measured in cell-free supernatants obtained by centrifugation at 250 x *g* for 5 min and immediately processed for ELISA, according to the manufacturer’s protocol. TNFα, IL-8 and IL-1β production was assessed by specific sandwich ELISA (Invitrogen, Thermo Fisher Scientific, Waltham, MA, USA; Immunotools GmbH, Friesoythe, Germany). Results were expressed as stimulation index. The limit of detection under optimal conditions was 4 pg/mL for TNFα, 2.6 pg/mL for IL-8, and 18 pg/mL for IL-1β.

### Plasmid DNA Preparation, Transient Transfections, and Luciferase Assay

Plasmids for transfections were purified with the HiSpeed^®^ Plasmid Midi Kit (Qiagen, Valencia, CA). DNA was quantified and assayed for purity using QuantusTM Fluorometer (Promega, Madison, WI). Transient transfections were performed in 12-multiwell culture plates; for each well 5 x 10^5^ cells were seeded in RPMI 1640 complete medium. Transfections were carried out using Lipofectamin 2000 Transfection Reagent (Invitrogen, Thermo Fisher Scientific, Waltham, MA, USA), according to manufacturer’s instructions. pGL4.32 vector (E8491, Promega, Madison, WI) luciferase-reporter construct plasmid DNA was co-transfected with pRL-TK Renilla (E2241, Promega, Madison, WI) luciferase expressing vector to measure transfection efficiency, as described in Buoso et al. (2019). During transfection THP-1 cells were incubated at 37°C in 5% CO_2_ overnight and, then, treated with 5 µM compounds and CURC for 24 h and, then, stimulated with 10 ng/mL LPS for 6 h. At the end of the treatments, cells were lysed with Passive Lysis Buffer provided by Dual-Luciferase^®^ Reporter Assay System, following manufacturer’s instructions (Promega, Madison, WI). The luminescent signals were measured using a 20/20 Luminometer with 10 s of integration (Turner BioSystems, Sunnyvale, CA).

### PBMCs Purification and Culture

Human peripheral blood mononuclear cells (PBMCs) were obtained from the blood of five (5) healthy individuals (mean age *±* SD: 71 ± 5.22 years; gender: 3 females and 2 males) satisfying the SENIEUR standard protocol for immuno-gerontological studies (Pawelec et al., 2001). Subjects having a history or physical signs of atherosclerosis or inflammation were excluded. All subjects gave written informed consent in accordance with the Declaration of Helsinki (Ethical Committee Project approval: Fulop_2019-2877). Heparinized blood was subjected to density gradient centrifugation over Ficoll-Paque Plus medium (GE Healthcare Life Sciences, Marlborough, MA, USA) as described in (Le Page et al., 2017). Briefly, PBS-diluted blood was carefully layered onto the Ficoll-Paque density gradient and centrifuged for 20 min at 400 x *g* at slow acceleration and with the brake off at room temperature. After centrifugation, the PBMCs layer, consisting of monocytes, T and B lymphocytes, was collected and washed three times with fresh PBS. Cell viability, assessed by Trypan blue exclusion, was more than 95%. For experiments, PBMCs were resuspended at a density of 1x10^6^ cells/mL in complete culture medium consisting of RPMI 1640 supplemented with 10% heat-inactivated FBS, 2 mM glutamine, 0.1 mg/mL streptomycin and 100 IU mL penicillin and maintained at 37°C in 5% CO_2_ and 95% air atmosphere.

### Luminex X-MAP^®^ Assay

Human cytokine MILLIPLEX^®^ MAP Kit (customized for IFNγ, IL-1β, IL-4, IL-6, IL-8, IL-12 (p40), IL-12 (p70), IL-13, IL-27, MCP-1, MCP-3, TNFα) was purchased from Millipore-Sigma (Merck KGaA). The assay was performed in a 96-well plate and all reagents were prepared according to the manufacturer’s instructions. Each well was cleaned and pre-wet with 200 μL of wash buffer on plate at 450 rpm during 10 min at RT. Wash buffer was removed by inverting the plate. Assay buffer, matrix solution or culture medium was used as a blank, each standard from a range of concentrations (different for each analyte), quality controls and samples were added to the appropriate wells. The mixed magnetic microbead solution was sonicated and vortexed prior to adding 25 μL into each well. The plates were sealed and incubated with agitation on a plate shaker at 750 rpm overnight at 4°C in a darkroom. Plates were put on the magnetic support to retain microbeads, then fluid was removed by inverting the plate to avoid touching the beads. Each well was washed three times with 200 μL of wash buffer with a plate shaker at 450 rpm for 30 s at RT. 25 μL of biotinylated detection antibodies were added per well, and plates were incubated in dark room at RT on a plate shaker at 750 rpm for 1 h. Then, 25 μL of streptavidin–phycoerythrin solution were added to each well, and plates were incubated on a plate shaker at 750 rpm for 30 min at RT and protected from light. Plates were washed three times with 200 μL of wash buffer. Microbeads were resuspended in 150 μL/well of sheath fluid on a plate shaker at 450 rpm for 5 min at RT. Data were acquired on a Luminex^®^ 200TM System using the Luminex xPonent^®^ software. An acquisition gate of between 8,000 and 15,000 was set to discriminate against any doublet events and ensure that only single microbeads were measured. Fifty beads/assay were collected and median fluorescence intensities (MFIs) were measured. Sensitivity limits (in pg/mL) were 0.86 for IFNγ; 0.52 for IL-1β; 0.2 for IL-4; 0.14 for IL-6; 0.52 for IL-8; 2.24 for IL-12 (p40); 0.88 for IL-12 (p70); 2.58 for IL-13; 50.78 for IL-27; 3.05 for MCP-1; 8.61 for MCP-3 and 5.39 for TNFα. MFIs were converted to concentrations using the equation of standard range of the appropriate cytokine using Milliplex^®^ Analyst 5.1 Software.

### Densitometry and Statistics

All the experiments were performed at least three times with representative results being shown. Data are expressed as mean ± SEM. The relative densities of the acquired images of Western blotting bands were analyzed with ImageJ software. Statistical analyses were performed using Prism software (GraphPad software, San Diego, CA, USA; version 8.0). Statistical differences were determined by analysis of variance (ANOVA) followed, when significant, by an appropriate *post hoc* test, as indicated in the figure legends. In all reported statistical analyses, effects were designated as non-significant for p > 0.05, significant (*) for *p* < 0.05 or less as indicated.

### Quantum Mechanics Calculations

The study for the conformational freedom of compounds 1–4 was conducted with the software Gaussian 09 (Gaussian Inc., Wallingford, CT, USA; Revision A.02). Each molecule underwent a protocol of geometrical optimization, involving an increasing level of precision of basis sets [i.e., from 3-21 (Binkley et al., 1980) to 6-31G* (Petersson and Al-Laham, 1991)], with the Hartree-Fock (HF) method (Kohn and Sham, 1965). The “Scan” functionality was used to estimate the barrier hindering conformational variability in the compounds for two different dihedral angles (**Supplementary Figure 2**). During this step, a Møller-Plesset correlation energy correction truncated at the second order (MP2) (Møller and Plesset, 1934) was added to the HF method and the torsions were rotated by intervals of 5 degrees until they completed the 360 degree turn. For each of these steps, the dihedral angle under study was fixed and the energy of the structure was computed after few steps of minimization.

## Results

### Cellular Toxicity of Compounds

**Figure 1:**
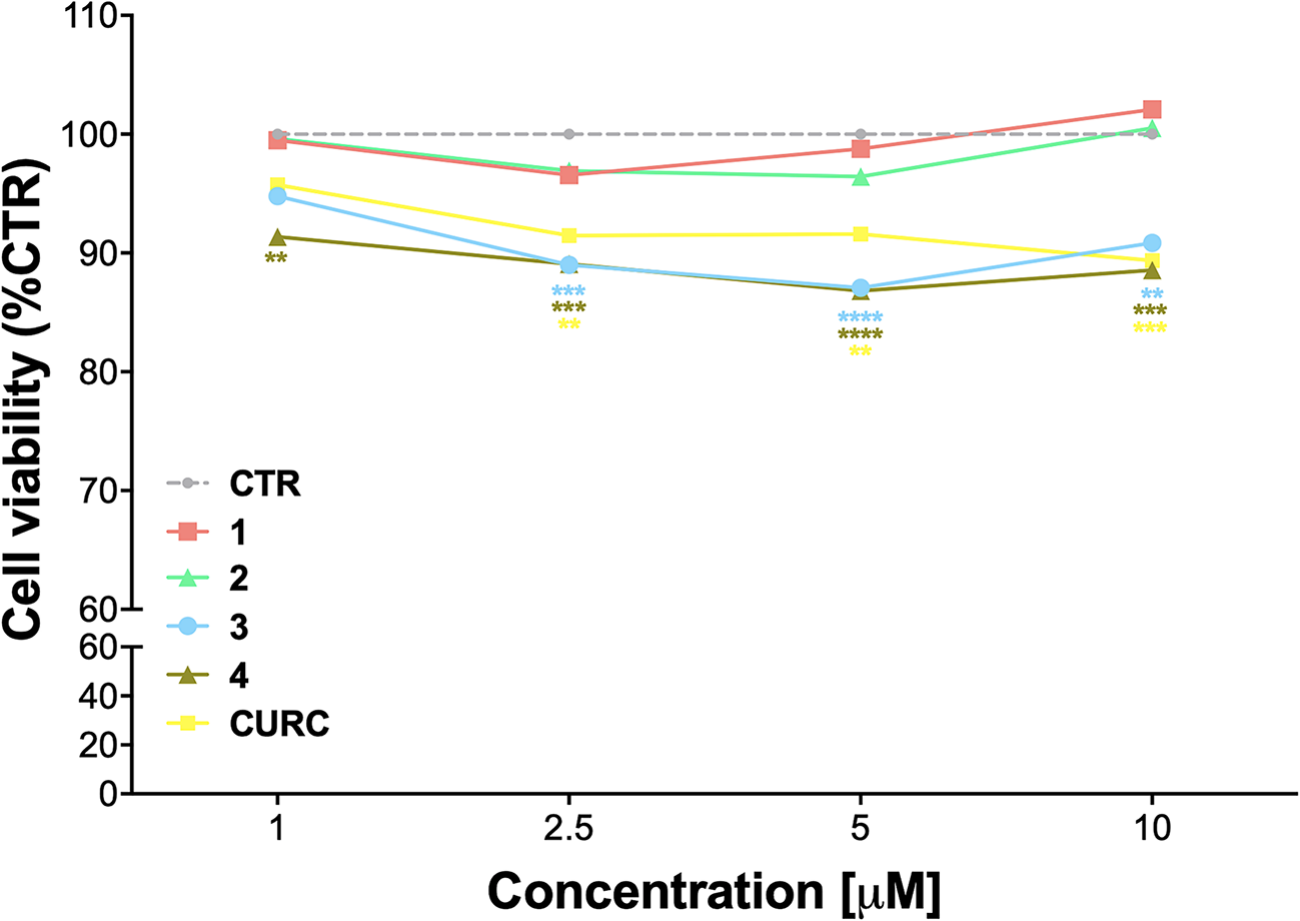
Cell viability in undifferentiated THP-1 exposed to compounds and CURC. THP-1 cells were treated with compounds 1*–*4 and CURC at the indicated concentrations for 24 h. Cell viability was assessed by MTT assay. Data are expressed as means of percentage of cell viability ± SEM. Dunnett’s multiple comparison test; **p < 0.01; ***p < 0.001 and ****p < 0.0001 *versus* CTR; n = 4.

The cytotoxicity of compounds 1–4 was assessed by MTT assay in THP-1 cells, in comparison with CURC, as a reference compound. Cells were exposed to compounds 1–4 and CURC at concentrations of 1 μM, 2.5 μM, 5 μM, and 10 μM for 24* h*. Consistently with our previous data on a different cellular model (Simoni et al., 2016; Simoni et al., 2017; Serafini et al., 2019; Catanzaro et al., 2020), all the compounds were well-tolerated, with a slight reduction of cell viability of about 10% observed for compounds 3 and 4 (**Figure 1**). Based on these results and according to our previous investigations (Simoni et al., 2017; Serafini et al., 2019; Catanzaro et al., 2020), all further experiments were conducted using the concentration of 5 μM.

### Modulation of Nrf2 Nuclear Translocation and HO-1 Target by Compounds

Nrf2 is a redox-sensitive transcription factor orchestrating the expression and coordinated induction of a wide battery of genes encoding phase II and detoxifying enzymes. Under unstressed conditions, Nrf2 is retained in the cytoplasm by its negative repressor Keap1 and rapidly subjected to ubiquitination and proteasomal degradation, mediated by the binding of Keap1 to the Cul3/Rbx1 E1 ubiquitin ligase complex (Niture et al., 2014). After exposure to oxidative and/or electrophilic stimuli, Nrf2 is released from structurally modified Keap1 and translocates into the nucleus, forms a heterodimer with one of the small musculoaponeurotic fibrosarcoma (Maf) proteins, and activates the ARE-mediated expression of cytoprotective genes. Since Nrf2 nuclear translocation is a fundamental step for the complete activation of its pathway, we tested the ability of compounds to induce the nuclear translocation of Nrf2 in THP-1 cells, by comparing their effects to CURC, used as a positive control.

Notably, evidence from the literature demonstrates that pro-electrophilic (catechol) and/or electrophilic moieties (the Michael acceptor α,β-unsaturated carbonyl group) are important structural functions necessary for the induction of the Nrf2 pathway (Tanigawa et al., 2007; Satoh et al., 2013). Compounds were synthesized and screened to identify the structural moieties responsible for the activation of Nrf2 and its downstream signaling pathway. The four compounds investigated in this study differ from each other by the presence or absence of the mentioned key functional groups (as shown in **Table 1**). Indeed, while compound 1 provides the catechol moiety, as well as the Michael acceptor group, 2 displays only the catechol moiety. Conversely, compounds 3 and 4 lack for both the Michael acceptor group and the catechol function.

**Figure 2:**
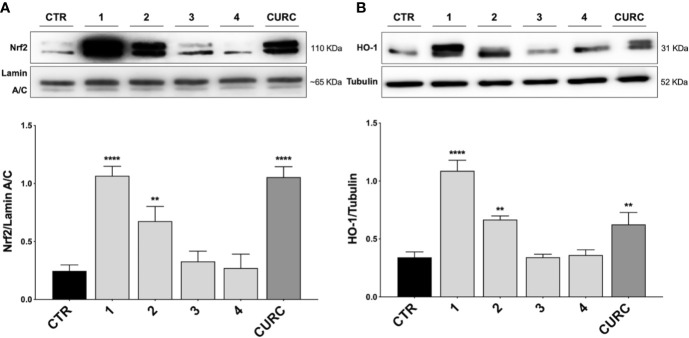
Nrf2 nuclear translocation and modulation of HO-1 protein content in THP-1 cells. **(A)** THP-1 cells were treated with compounds 1*–*4 and CURC at a concentration of 5 μM for 3 h. After isolation, nuclear extracts were examined by Western blot analysis and Nrf2 expression was determined using an anti-Nrf2 antibody. Anti-lamin A/C was used as protein loading control. Results are shown as means of Nrf2/lamin A/C ratio ± SEM. Dunnett’s multiple comparison test; **p < 0.01 and ****p < 0.0001 *versus* CTR; n = 5–7. **(B)** Total protein extracts of THP-1 cells, treated with compounds 1*–*4 and CURC at the concentration of 5 μM for 24 h, were analyzed for HO-1 protein content by Western blot analysis. Anti-tubulin was used as protein loading control. Results are shown as means of HO-1/Tubulin ratio ± SEM. Dunnett’s multiple comparison test; **p < 0.01 and ****p < 0.0001 *versus* CTR; n = 7.

Thus, THP-1 cells were treated with DMSO as vehicle control, compounds 1–4 and CURC at a concentration of 5 μM for 3* h*. After treatment, Nrf2 nuclear content was assessed by Western blot analysis. As shown in **Figure 2A**, compounds 1, 2, as well as CURC, significantly induced Nrf2 nuclear translocation, whereas compounds 3 and 4 did not increase Nrf2 nuclear content (**Figure 2A**). Such results are consistent with our previous work (Simoni et al., 2017; Serafini et al., 2019; Catanzaro et al., 2020), where the ability of compounds 1 and 2, but not 3, to activate the Nrf2 pathway in SH-SY5Y and ARPE-19 cells suggested that the addition of Keap1 nucleophilic cysteines to (pro)electrophilic portions of the molecule could represent the initiating event. The finding that the newly synthesized molecule, compound 4, was also unable to induce Nrf2 nuclear translocation corroborates this hypothesis.

To demonstrate the downstream activation of the Nrf2 signaling pathway, the protein amount of HO-1, one of the main targets of Nrf2, was evaluated by Western blot analysis. THP-1 cells were treated with DMSO as vehicle control, compounds 1–4 and CURC at a concentration of 5 μM for 24* h*. As shown in **Figure 2B**, compounds 1, 2 and CURC positively modulated HO-1 protein levels, confirming the activation of the Nrf2 pathway. In contrast, compounds 3 and 4 did not affect the protein amount of HO-1 in THP-1 whole cell lysates, confirming their inability to promote Nfr2 pathway activation.

### Compounds Attenuate TNFα and IL-1β, but Not IL-8 Release, in LPS-Stimulated THP-1 Cells

**Figure 3:**
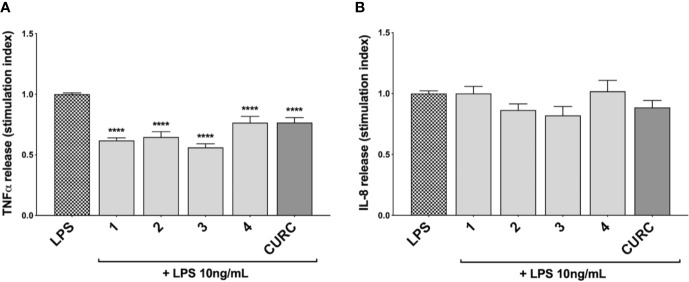
Modulation of TNFα and IL-8 release in LPS-stimulated THP-1 cells exposed to compounds. THP-1 cells were treated with compounds 1*–*4 and CURC at a concentration of 5 μM for 24 h, and then stimulated with 10 ng/mL LPS for 3 h. TNFα **(A)** and IL-8 **(B)** protein release was measured in THP-1 supernatants by ELISA. Data are presented as means of stimulation index ± SEM. Dunnett’s multiple comparison test; ****p < 0.0001 *versus* CTR; n = 5.

**Figure 4:**
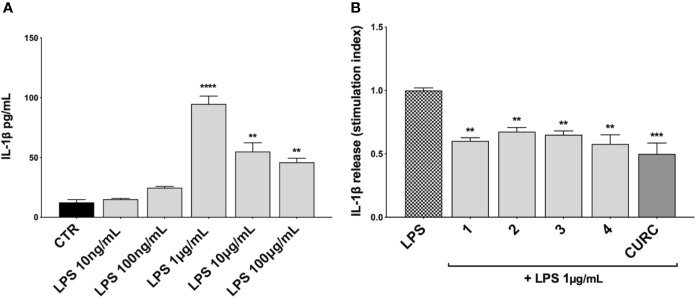
Modulation of IL-1β release in LPS-stimulated THP-1 cells. **(A)** IL-1β protein secretion was measured in THP-1 cell supernatants stimulated with LPS at the indicated concentrations for 3 h. At the end of the treatment, IL-1β protein release was assessed by ELISA. Data are presented as means of released picograms per mL (pg/mL) ± SEM. Dunnett’s multiple comparison test; **p < 0.01 and ****p < 0.0001 *versus* CTR; n = 3. **(B)** IL-1β protein release was measured in THP-1 cells supernatants, treated for 24 h with compounds 1*–*4 and CURC at a concentration of 5 μM and then stimulated with 1 μg/mL LPS for 3 h. The level of IL-1β was assessed by ELISA. Data are presented as means of stimulation index ± SEM. Dunnett’s multiple comparison test; **p < 0.01 and ***p < 0.001 *versus* CTR; n = 3.

To investigate the immunomodulatory potential of compounds acting as Nrf2 inducers, we exposed THP-1 cells to LPS from *E. coli*, resulting in enhanced production and secretion of pro-inflammatory mediators (**Supplementary Figure 3** and **Figure 4A**). Thus, THP-1 cells were treated with DMSO as vehicle control, compounds 1–4 and CURC at a concentration of 5 μM for 24* h* and, then, exposed to 10 ng/mL LPS for 3* h* in order to evoke the inflammatory response (**Figure 3**). TNFα (**Figure 3A**) and IL-8 protein release (**Figure 3B**) were measured by ELISA in the supernatants of LPS-stimulated THP-1 cells. Notably, all compounds, independently from their ability to act as Nrf2 inducers, significantly reduced TNFα protein release into cell culture medium (**Figure 3A**). In contrast, in the same experimental setting, all compounds, as well as CURC, did not affect IL-8 protein release into cell culture medium (**Figure 3B**).

We further investigated the effects of compounds 1–4 and CURC on the release of the pro-inflammatory mediator IL-1β upon stimulation. Unlike TNFα and IL-8, no increase in IL-1β protein release was observed in THP-1 cells exposed to 10 ng/mL LPS, but only after stimulation with 1 μg/mL LPS for 3* h*, as reported in **Figure 4A**. Then, THP-1 cells were treated with DMSO as vehicle control, compounds 1–4 and CURC at a concentration of 5 μM for 24* h*, exposed to 1 μg/mL LPS for 3* h* to promote the inflammatory response, and tested for IL-1β release by ELISA (**Figure 4B**). All the compounds, as well as CURC, significantly reduced IL-1β protein release into cell culture medium. As observed for TNFα, both compounds acting as Nrf2 inducers (1 and 2) and those inactive on the Nrf2 pathway (3 and 4) counteracted the LPS-driven inflammatory response, thus suggesting the involvement of different intracellular pathways.

### siRNA Mediated Nfr2 Knockdown Does Not Affect TNFα Release in LPS-Stimulated THP-1 Cells

**Figure 5:**
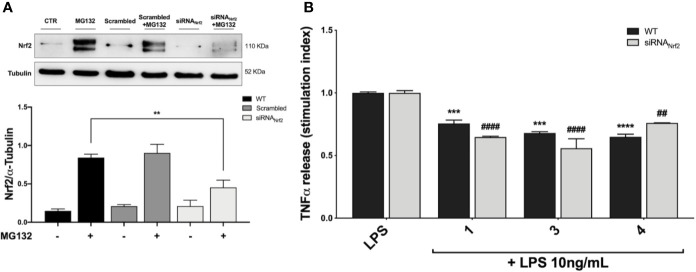
Optimization of Nfr2-silenced THP-1 model **(A)** and effect of Nrf2-knockdown on modulation of TNFα release by compounds 1, 3 and 4, upon LPS stimulation **(B)**. **(A)** THP-1 cells were treated either with vehicle (WT), scrambled or siRNA_Nrf2_ for 24 h. Where indicated MG132 was added 4 h before the end of the experiment to block the proteasomal degradation of Nrf2. After treatments, Nrf2 expression was determined in total protein extracts by Western blot analysis using an anti-Nrf2 antibody. Anti-α-tubulin was used as protein loading control. Results are shown as means of Nrf2/α-Tubulin ratio ± SEM. Unpaired Student *t*-test; **p < 0.01; n = 3. **(B)** TNFα amount was measured in the supernatants of THP-1 Nrf2-knockdown cells, treated with compounds 1, 3, and 4 at a concentration of 5 μM for 24 h and then stimulated with 10 ng/mL LPS for 3 h. The protein secretion of TNFα was determined by ELISA. Data are shown as means of stimulation index ± SEM. Dunnett’s multiple comparison test; ***p < 0.001 and ****p < 0.0001 *versus* WT LPS; ^##^*p* < 0.01 and ^####^*p* < 0.0001 *versus* siRNA_Nrf2_ LPS; n = 3.

Based on the effect elicited by compounds 3 and 4 on cytokine release, the Nrf2 gene was knocked down by siRNA in THP-1 cells with the aim to evaluate the weight of the Nrf2 pathway in pro-inflammatory cytokine modulation upon LPS stimulation. Accordingly, cells were transfected with scrambled (siRNA_CTR_) and Nrf2 siRNA (siRNA_Nrf2_) for 24* h* and the proteasome inhibitor MG132 (5 µM) was added 4* h* before the end of the experiment to the medium of selected plates in order to assess Nrf2 silencing. Nrf2 shows a short half-life, with a rapid ubiquitin-proteasome-mediated degradation (Kobayashi and Yamamoto, 2006). Thus, to properly appreciate Nrf2 silencing, we blocked Nrf2 degradation using the proteasome inhibitor MG132. After treatments, the Nrf2 protein content was measured in whole cell lysates by Western blot analysis. As reported in **Figure 5A**, the proteasome inhibitor MG132 induced an increase in Nrf2 protein levels in comparison with control. No statistically significant difference in Nrf2 protein levels between wild type (WT) and scrambled treated cells, treated with MG132, was found, whereas a marked decrease in Nrf2 protein content between WT and siRNA_Nrf2_-treated cells was observed (**Figure 5A**). Then, WT and siRNA_Nrf2_ cells were treated for 24* h* with 5 μM of selected compounds (the Nrf2 inducer 1, and the inactive 3 and 4), stimulated with 10 ng/mL LPS for 3* h* to evoke the inflammatory response, and analysed for TNFα release by ELISA. Notably, all the selected compounds significantly suppressed LPS-induced release of TNFα both in WT and siRNA_Nrf2_ cells (**Figure 5B**), indicating that the observed reduction in pro-inflammatory cytokines release upon LPS stimulation, cannot be explained on the basis of the activation of Nrf2 pathway.

### Modulation of the NF-κB Cellular Pathway by Compounds

**Figure 6:**
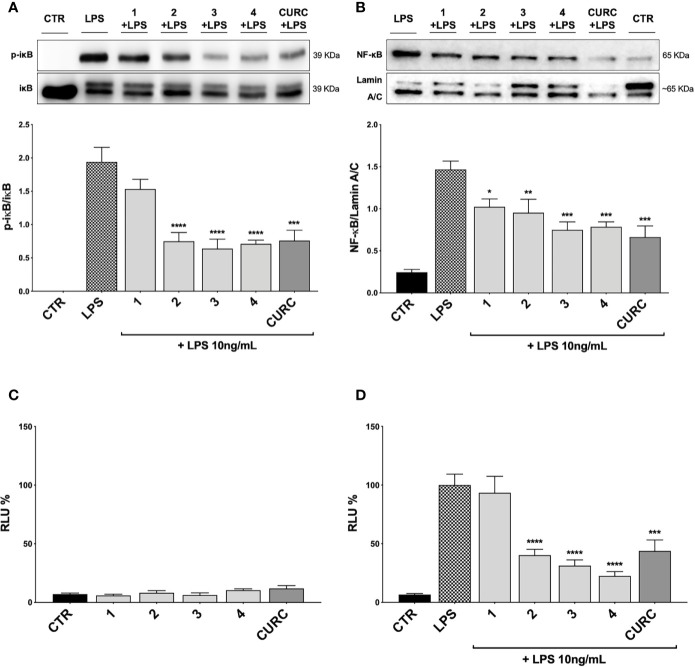
Modulation of NF-κB pathway by compounds and CURC in LPS-stimulated THP-1 cells. **(A)** THP-1 cells were treated with 5 μM compounds 1*–*4 and CURC for 24 h and then stimulated with 10 ng/mL LPS for 45 min. After stimulation, p-IκBα expression was determined in total protein extracts by Western blot analysis, using an anti-p-IκBα antibody. Anti-IκBα (total) was used to normalize the data. Results are shown as means of p-IκBα/IκBα ratio ± SEM. Dunnett’s multiple comparison test; ***p < 0.001 and ****p < 0.0001 *versus* LPS; n = 5. **(B)** THP-1 cells were treated for 24 h with compounds 1*–*4 and CURC at a concentration of 5 μM and then stimulated with 10 ng/mL LPS for 90 min. After isolation, nuclear extracts were examined by Western blot analysis and NF-κB expression was determined using an anti-NF-κB antibody. Anti-lamin A/C was used as protein loading control. Results are shown as means of NF-κB/Lamin A/C ratio ± SEM. Dunnett’s multiple comparison test; *p < 0.05, **p < 0.01 and ***p < 0.001 *versus* LPS; n = 5. **(C, D)** THP-1 cells were transiently transfected with pGL4.32 [luc2P/NF-κB-RE/Hygro] Vector reporter construct, and subsequently treated with compounds 1*–*4 and CURC at a concentration of 5 μM for 24 h. After treatments, the cells were stimulated **(D)** or not **(C)** with 10 ng/mL LPS for 6 h. For each condition, luciferase activity was expressed as RLU% and compared to CTR values assumed at 100%. Results are shown as means ± SEM. Dunnett’s multiple comparison test; ***p < 0.001 and ****p < 0.0001 *versus* LPS; n = 3.

To better understand the mechanism of action underlying the reduction of cytokines induced by compounds, we investigated their potential effect on the NF-κB pathway. Exposure of THP-1 cells to LPS from *E. coli* resulted in activation of the NF-κB transcription factor (Gomes et al., 2015; Sakai et al., 2017). To assess the effect of compounds on the NF-κB signaling pathway, we investigated the modulation of the upstream signaling molecule IκBα. In our experimental setting, THP-1 cells were treated with DMSO as vehicle control, compounds 1–4 and CURC at a concentration of 5 μM and, then, stimulated for 45* min* with 10 ng/mL LPS. The phosphorylation of IκBα was measured in whole cell lysates by Western blot analysis. As shown in **Figure 6A**, LPS stimulation markedly increased the level of p-IκBα compared to controls, whereas treatments with compounds 2, 3, 4, and CURC significantly prevented IκBα phosphorylation, thus indicating that they might hinder the activation of the NF-κB pathway by preventing IκBα phosphorylation. Compound 1 did not produce statistically significant results in our experimental setting, although a slight trend to decrease in IκBα phosphorylation could be observed (**Figure 6A**).

To further evaluate the capability of compounds to influence NF-κB nuclear translocation, THP-1 cells were treated with vehicle, 5 μM compounds 1–4 and CURC and, then, stimulated for 1* h* and 30* min* with 10 ng/mL LPS, as inflammatory stimulus promoting NF-κB nuclear translocation. Compounds 3 and 4, and CURC markedly suppressed NF-κB nuclear translocation, whereas compound 2 acted to a lower extent (**Figure 6B**). Compound 1 did not produce statistically significant results in our experimental setting, although a slight trend to decrease could be observed (**Figure 6B**).

Finally, we investigated the activation of the NF-κB promoter by luciferase assay. To evaluate whether compounds may exert a basal activity on NF-κB promoter, THP-1 cells were transiently transfected with pGL4.32 luciferase reporter construct, containing NF-κB-response elements (RE), and treated with vehicle, 5 μM compounds 1–4 and CURC for 24* h* and, then, analyzed for NF-κB luciferase. No difference in NF-κB luciferase activity between untreated and treated cells was observed, thus suggesting that compounds did not basally influence NF-κB pathways (**Figure 6C**). THP-1 cells were then stimulated with 10 ng/mL LPS for 6* h* after treatment with vehicle and 5 μM compounds 1–4 and CURC for 24* h*. As reported in **Figure 6D**, upon LPS stimulation, NF-κB luciferase activity significantly increased, as expected, and treatments with compounds 2, 3, 4, and CURC significantly reduced it. In accordance with the slight effect on IκBα phosphorylation and NF-κB nuclear translocation, compound 1 did not hinder the activation of the NF-κB promoter, thus suggesting that the modulation of cytokine release by this molecule seems not to be driven by NF-κB signaling pathway (**Figure 6D**).

### Differential Regulation of Innate Immune Cytokine Release in Human PBMCs From Healthy Donors

To further study the differential capability of compounds in modulating cytokine and chemokine release, we moved from THP-1 cells to human primary PBMCs from healthy donors. Human PBMCs were stimulated with 10 ng/mL LPS for 3* h* after having been treated with vehicle and 5 µM compounds 1–4 and CURC for 24* h*. The release of a panel of the most common cytokines and chemokines [e.g. IFNγ, IL-1β, IL-4, IL-6, IL-8, IL-12 (p40), IL-12 (p70), IL-13, IL-27, MCP-1, MCP-3, TNFα] was measured in culture medium by Luminex X-MAP*^®^* technology. Protein release of IFNγ, IL-4, IL-12 (p70), IL-13 and IL-27 was undetectable both in untreated and LPS-stimulated PBMCs from healthy donors, while, exposure of human PBMCs to LPS significantly increased protein release of IL-6, IL-8, IL-12 (p40), MCP-1, and TNFα compared to controls (**Table 2**). A differential regulation of cytokine and chemokine release by compounds was observed during immune stimulation. In particular, compounds 1 and CURC, significantly reduced the release of the pro-inflammatory cytokine IL-6 in LPS-stimulated human PBMCs. In accordance with preliminary results obtained in THP-1 cells, no effect on IL-8 release was observed for 1–4 and CURC, further indicating that all compounds, as well as CURC, did not influence the intracellular pathways regulating IL-8 release. In addition, compounds 1 and 2 significantly decreased IL-12 (p40) release in human PBMCs upon LPS stimulation (**Table 2**). No differences in IL-1β and MCP-3 release were observed between PBMCs that were stimulated by LPS, untreated, or treated with compounds or CURC PBMCs (**Table 2**). Interestingly, compounds 1, 2, and CURC were capable to significantly attenuate the release of the chemokine MCP-1 in LPS-stimulated PBMCs from healthy patients. In contrast, compounds 3 and 4 did not affect MCP-1 release, revealing the same activity trend observed for Nrf2 induction (**Table 2**). Notably, such results are consistent with evidence from literature reporting that, after innate immune stimulation, treatment of human PBMCs with Nrf2 activators, such as the Nrf2 agonist CDDO-Me (bardoxolone methyl), markedly reduced LPS-evoked MCP-1/CCL2 production and that this effect was not specific to LPS-induced immune responses, as Nrf2 activation also reduced MCP-1/CCL2 production after stimulation with IL-6 (Eitas et al., 2017). Furthermore, compound 4 and CURC confirmed their capability to significantly reduce TNFα release in LPS-stimulated PBMCs, as previously observed in the THP-1 cell line (**Table 2**).

**Table 2:** Differential regulation of innate immune cytokine release in human PBMCs from healthy donors.

Luminex xMAP^®^ Technology							
	CTR	LPS10 ng/mL	1+ LPS	2+ LPS	3+ LPS	4+ LPS	CURC+ LPS
IFNγ	Nd	Nd	Nd	Nd	Nd	nd	nd
IL-1β	2.76 ± 0.56	2.43 ± 0.49	1.36 ± 0.14	1.58 ± 0.16	1.85 ± 0.30	2.59 ± 0.56	1.31 ± 0.17
IL-4	Nd	Nd	nd	Nd	Nd	nd	nd
IL-6	33.20 ± 6.38****	164.4 ± 12.1	129.2 ± 7.68*	139.9 ± 8.32	147.1 ± 10.5	156.0 ± 11.4	99.65 ± 6.71****
IL-8	1659 ± 178****	3349 ± 292	4012 ± 141	3272 ± 224	2794 ± 124	3026 ± 333	2665 ± 221
IL-12 (p40)	2.44 ± 0.14**	5.56 ± 0.57	2.40 ± 0.12**	3.28 ± 0.27*	4.82 ± 0.87	5.30 ± 0.90	3.61 ± 0.70
IL-12 (p70)	nd	Nd	nd	nd	Nd	nd	nd
IL-13	nd	Nd	nd	nd	Nd	nd	nd
IL-27	nd	Nd	nd	nd	nd	nd	nd
MCP-1	1149 ± 89.2*	1627 ± 139	899 ± 163***	819 ± 89.5****	1243 ± 125	1198 ± 105	625 ± 76.9****
MCP-3	38.9 ± 8.2	50.6 ± 8.9	36.7 ± 6.0	35.11 ± 5.7	43.0 ± 8.3	42.1 ± 6.9	25.3 ± 3.3
TNFα	13.75 ± 2.76****	350.2 ± 18.6	264.8 ± 6.74	295.2 ± 18.7	328.3 ± 16.3	220.8 ± 31.1**	252.8 ± 38.6*

PBMCs were treated with compounds 1–4 and CURC at a concentration of 5 μM for 24 h, and then stimulated with 10 ng/mL LPS for 3 h. IFNγ, IL-1β, IL-4, IL-6, IL-8, IL-12 (p40), IL-12 (p70), IL-13, IL-27, MCP-1, MCP-3, TNFα protein release was measured in PBMC supernatants by Luminex xMAP^®^ Technology. Data are presented as means of released picograms per mL (pg/mL) ± SEM. Dunnett’s multiple comparison test; *p < 0.05; **p < 0.01; ***p < 0.001 and ****p < 0.0001 versus LPS; n = 5.

## Discussion

The transcription factor Nrf2 regulates a complex network of cellular responses to oxidative stress and inflammation. Cysteine residues of its repressor Keap1 act as sensor sites for Nrf2 electrophilic activators. Thus, we studied a set of previously synthesized compounds (1, 2, and 3), for which the ability to induce the Nrf2 pathway was strictly related to the (pro)-electrophilic character of the molecule in THP-1 cells, a widely used cellular model for the immune modulation approach (Chanput et al., 2014). In agreement with previous results (Simoni et al., 2017; Serafini et al., 2019; Catanzaro et al., 2020), a significant effect was detected for the Nrf2 inducers 1 and 2, carrying a catechol moiety and/or an α,β-unsaturated carbonyl group, while no effect was observed for compound 3, lacking both (pro)-electrophilic features (**Table 1**). The same lack of effect was observed for compound 4, which was included in the study to exclude possible oxidative activation into electrophilic metabolites such as quinone methide, which could provide an additional site for adduct formation.

**Figure 7:**
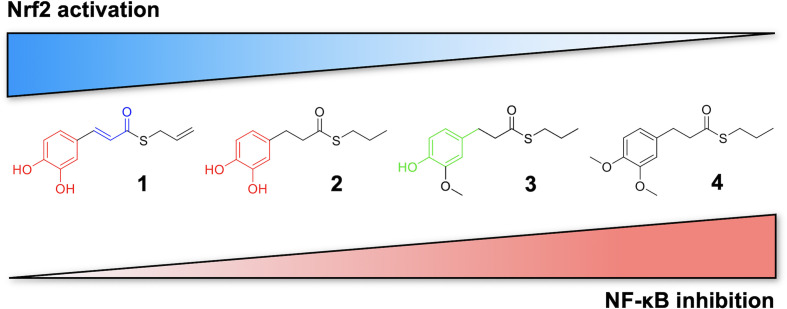
Differential modulation of Nrf2 and NF-κB intracellular signaling pathways by compounds. Electrophile 1, carrying both the catechol moiety (red) and the α,β-unsaturated carbonyl group (blue), is the most active Nrf2 inducer, while being devoid of activity on NF-kB pathway. Conversely, the non-electrophilic compound 4, synthesized to exclude eventual oxidative transformation of the methoxyphenol ring (green) of 3 into reactive metabolites, is the most potent NF-kB inhibitor, with no impact on Nrf2 activation.

**Figure 8:**
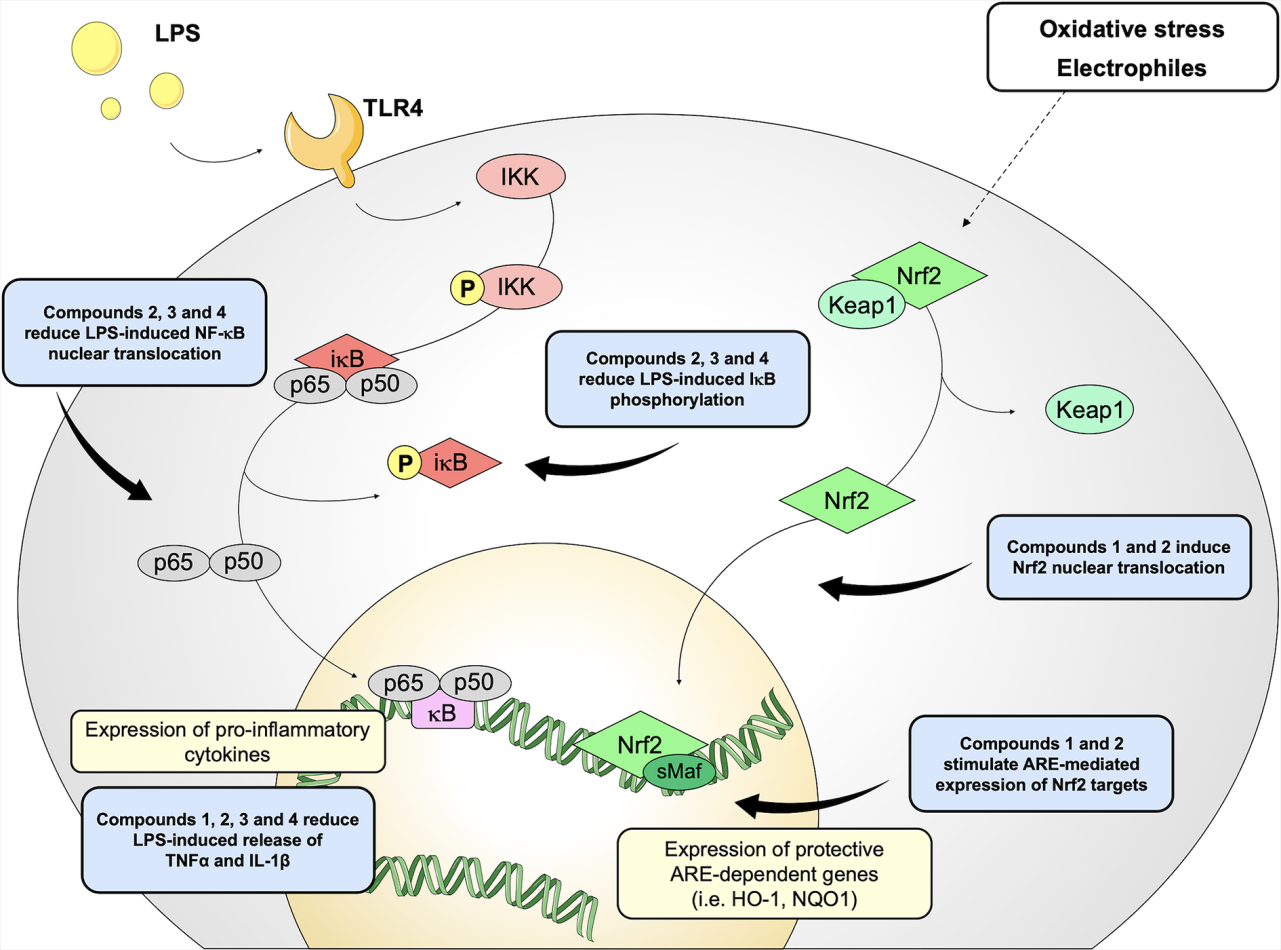
Schematic representation of the effects induced by compounds 1*–*4 on Nrf2 and NF-κB pathways.

Based on these results, we investigated the potential effects of the compounds on the secretion of pro-inflammatory cytokines upon immune stimulation (e.g. LPS from *E. coli*) in the same cellular model. We found that both compounds which induced Nrf2 (1 and 2) as well as compounds inactive on the Nrf2 pathway (3 and 4) were capable to attenuate the release of the pro-inflammatory cytokines TNFα (**Figure 3A**) and IL-1β (**Figure 4B**), but not IL-8 secretion (**Figure 3B**), thus suggesting that the reduction of cytokine release by compounds could not be directly ascribed to the activation of Nrf2 pathway. Accordingly, the ability of compounds to attenuate the secretion of TNFα, upon immune stimulation, was also observed after siRNA mediated Nrf2 knockdown (**Figure 5**). To further dissect the molecular mechanism underlying the reduction of cytokine release induced by compounds 1*–*4, we investigated their potential interplay with other signaling cascades, specifically focusing on the NF-κB pathway, a pivotal mediator of inflammatory responses and critical regulator of multiple aspects of innate and adaptative immune functions (Häcker and Karin, 2006; Perkins, 2007). All compounds, with the exception of compound 1, significantly attenuated the LPS-induced activation of the NF-κB canonical pathway, by impairing the upstream phosphorylation of IκBα, NF-κB nuclear translocation, as well as the activation of the NF-κB promoter (**Figure 5**). As a consequence, the ability of compounds 2, 3, and 4 to reduce the activation of NF-κB pathway may account, at least in part, for their observed effect on pro-inflammatory cytokine release. Notably, both Nrf2 and NF-κB offer unique patterns of thiol modifications, indicating electrophilic signaling mediators as a valuable instrument to control their redox-sensitive transcriptional regulatory function. However, while a (pro)-electrophilic feature is required for Nrf2 induction, suggesting covalent adduction as the triggering event, both (pro)-electrophile 2 and non-electrophilic compounds 3 and 4 were able to inhibit NF-κB activation, revealing a different mode of interaction (**Figure 7**). Noteworthy, compound 1, carrying both the catechol moiety and the α,β-unsaturated carbonyl group, was unable to significantly modulate the NF-κB pathway. The modulation of cytokine release by this molecule might be, at least in part, related to anti-inflammatory effect mediated by the induction of Nrf2 targets, such as HO-1 (Roach et al., 2009). Accordingly, HO-1 expression has been demonstrated to decrease the LPS-stimulated secretion of cytokines and chemokines such as MCP-1, IL-6, IL-10, and TNFα in murine and human macrophages (Roach et al., 2009). Altogether, these results indicate that an electrophilic moiety is neither necessary nor per se sufficient to guarantee inhibition of the pro-inflammatory transcriptional activity of NF-κB, with shape complementarity emerging as a plausible feature of target recognition. The different biological behavior of electrophiles 1 and 2, which only varies in the presence or absence of two double bonds, might indeed reflect the more constrained conformation assumed by compound 1 with respect to flexible compound 2. The conjugation extended throughout most of the backbone stabilized compound 1 in a planar conformation, as opposed to the sp3 counterparts which manifested a maximum in energy for the same state (**Supplementary Figure 2**). Moreover, the possibility for compounds 2-4 to populate several conformations due to lower energy barriers might indicate that a conformational selection or induced fit effect in the ligand is necessary to execute the desired activity. The overall effects of compounds 1*–*4 on Nrf2 and NF-κB intracellular pathways were summarized in **Figure 8**.

When moving in a human primary model, by Luminex X-MAP**^®^** technology, we screened the effects of compounds on a panel of cytokines and chemokines (e.g. IFNγ, IL-1β, IL-4, IL-6, IL-8, IL-12 (p40), IL-12 (p70), IL-13, IL-27, MCP-1, MCP-3, TNFα) in order to unveil their potential modulatory effect on other inflammatory mediators (**Table 2**). Compared to data observed in THP-1 cells, we found a differential regulation of innate immune cytokine production by compounds, in line with previous data (Schildberger et al., 2013). In particular, compounds 1 and CURC significantly reduced the secretion of the pro-inflammatory cytokine IL-6 in LPS-stimulated human PBMCs, while compound 4 and CURC attenuated TNFα release (**Table 2**). Furthermore, compounds acting as Nrf2 inducers (1 and 2) also suppressed the secretion of IL-12 (p40), corroborating the hypothesis that inhibition of IL-12 expression may be mediated by Nrf2 activation, as suggested by Macoch et al. (2015). Consistently, tert-butylhydroquinone, a well-known Nrf2 inducer, has been reported to activate Nrf2 and to inhibit the induction of IL-12 expression by LPS (Macoch et al., 2015). However, further investigations are required to unravel the molecular mechanism by which Nrf2 represses IL-12 production and secretion.

In human PBMCs, we further found that only compounds acting as Nrf2 inducers (1 and 2) significantly suppressed the release of MCP-1, after LPS stimulation (**Table 2**). In accordance with data from the literature (Eitas et al., 2017), this result indicates that MCP-1 production may rely on activation of the transcription factor Nrf2. Thus, the effect of Nrf2 inducers on MCP-1/CCL2 suggests a novel aspect of Nrf2 pharmacological activation as a regulator of key immunomodulatory functions. This finding represents a potentially generalizable aspect of pharmacological Nrf2 activation occurring with different stimuli (e.g. LPS, IL-6) and consistent across more than 60 individual human samples, as reported by Eitas et al. (2017). Thus, contrary to the prevalent view that Nrf2 represses inflammatory processes through redox control, we demonstrated that Nrf2 activation also directly counteracts the production of a key chemokine, by possibly regulating the expression of its encoding gene. Such hypothesis is consistent with data reporting Nrf2-mediated downregulation of proinflammatory mediator gene expression (Kobayashi et al., 2016). However, the precise molecular mechanism underlying Nrf2 and MCP-1 crosstalk is still elusive. Interestingly, by regulating the production of the chemokine MCP-1, Nrf2 can be considered an upstream regulator of MCP-1 production, thereby providing a molecular basis for a Nrf2-mediated anti-inflammatory approach. In this regard, elevated systemic MCP-1 system levels have been linked to worse outcomes in patients with cardiovascular disease (Martín-Ventura et al., 2009), and pulmonary accumulation of MCP-1 has been reported in patients with acute respiratory distress syndrome (Rosseau et al., 2000). Hence, targeting transcriptional accumulation of MCP-1 through pharmacological Nrf2 activation may represent a promising therapeutic approach.

Although the THP-1 and human PBMCs response can hint to potential responses that may occur *in vivo*, these results need to be validated in *in vivo* studies to draw more definite conclusions. Moreover, further mechanistic investigations are required to unravel the biological connection among Nrf2 activation, innate immune cytokine production, and the regulation of the NF-κB pathway.

## Data Availability Statement

The raw data supporting the conclusions of this article will be made available by the authors, without undue reservation, to any qualified researcher.

## Ethics Statement

The studies involving human participants were reviewed and approved by Ethical Committee Project approval: Fulop_2019-2877. The patients/participants provided their written informed consent to participate in this study.

## Author Contributions

Conceived and designed the experiments: FF, MC, EC, MRo, and CL. Chemical synthesis: FB and MRo. Performed the experiments and analyzed the data: FF, MC, and EB. Quantum mechanics data: DM and SR. Critical discussion: FF, MC, EC, MRa, MA, TF, SG, MRo, and CL. Funding acquisition: EF, TP, EC, MRa, MRo, and CL.

## Funding

Research has been supported by the University of Pavia (grants from FR&G 2019, Fondo Ricerca & Giovani, to CL; PRIN 2017B9NCSX_003 to MRa; an Educational Grant from Aboca S.p.A. to MRa and SG), the University of Milan (grants from PRIN 2017MLC3NF to EC), the University of Bologna (grants from the RFO to MRo) and grants from Université de Sherbrooke, FRQS “AUDACE” program and the Research Center of the CHUS to EF and TF).

## Conflict of Interest

The authors declare that the research was conducted in the absence of any commercial or financial relationships that could be construed as a potential conflict of interest.
