# Supplementary figures and images for: Targeting Cytokine Release Through the Differential Modulation of Nrf2 and NF-κB Pathways by Electrophilic/Non-Electrophilic Compounds
Source: Front Pharmacol. 2020 Aug 14;11:1256. doi: 10.3389/fphar.2020.01256 (PMC7456937; doi:10.3389/fphar.2020.01256)

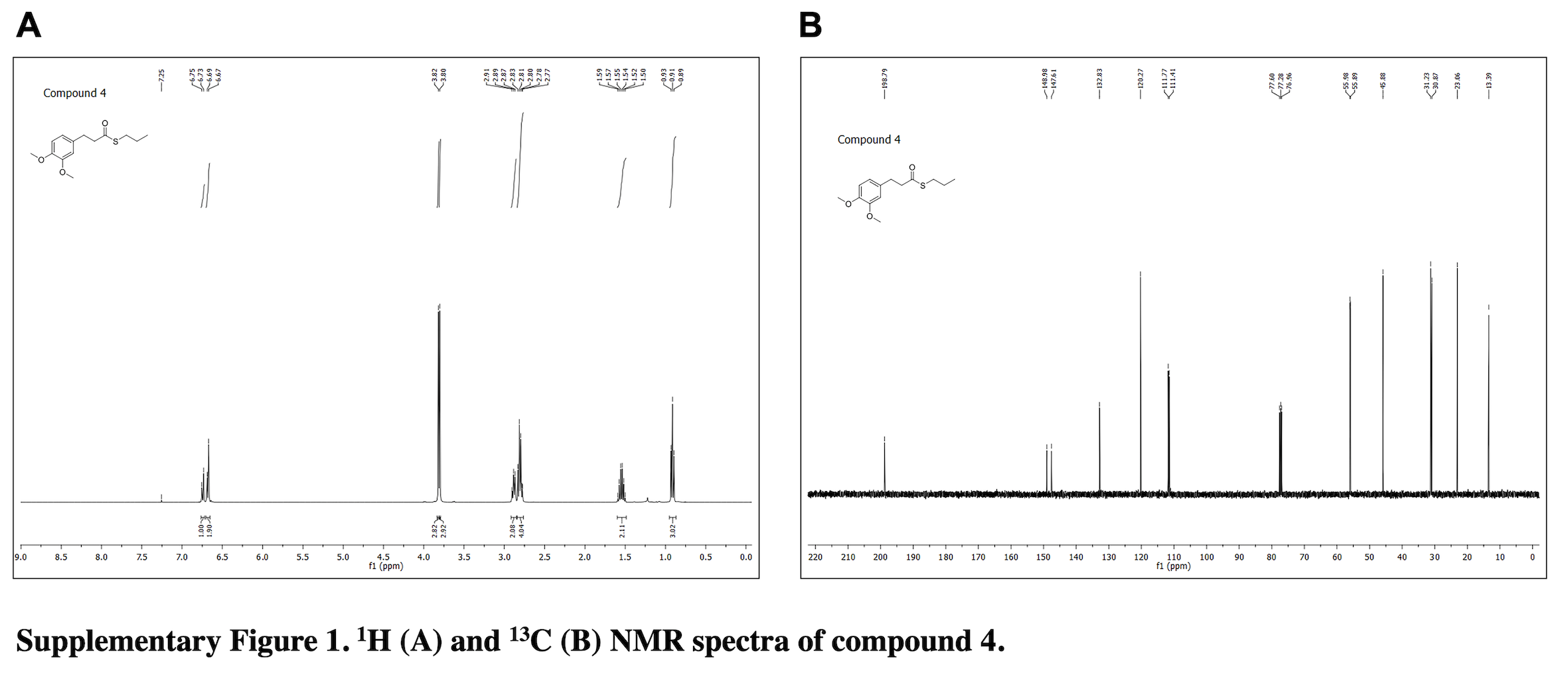

Supplement: Supplementary file 1 [file Image_1.tif]

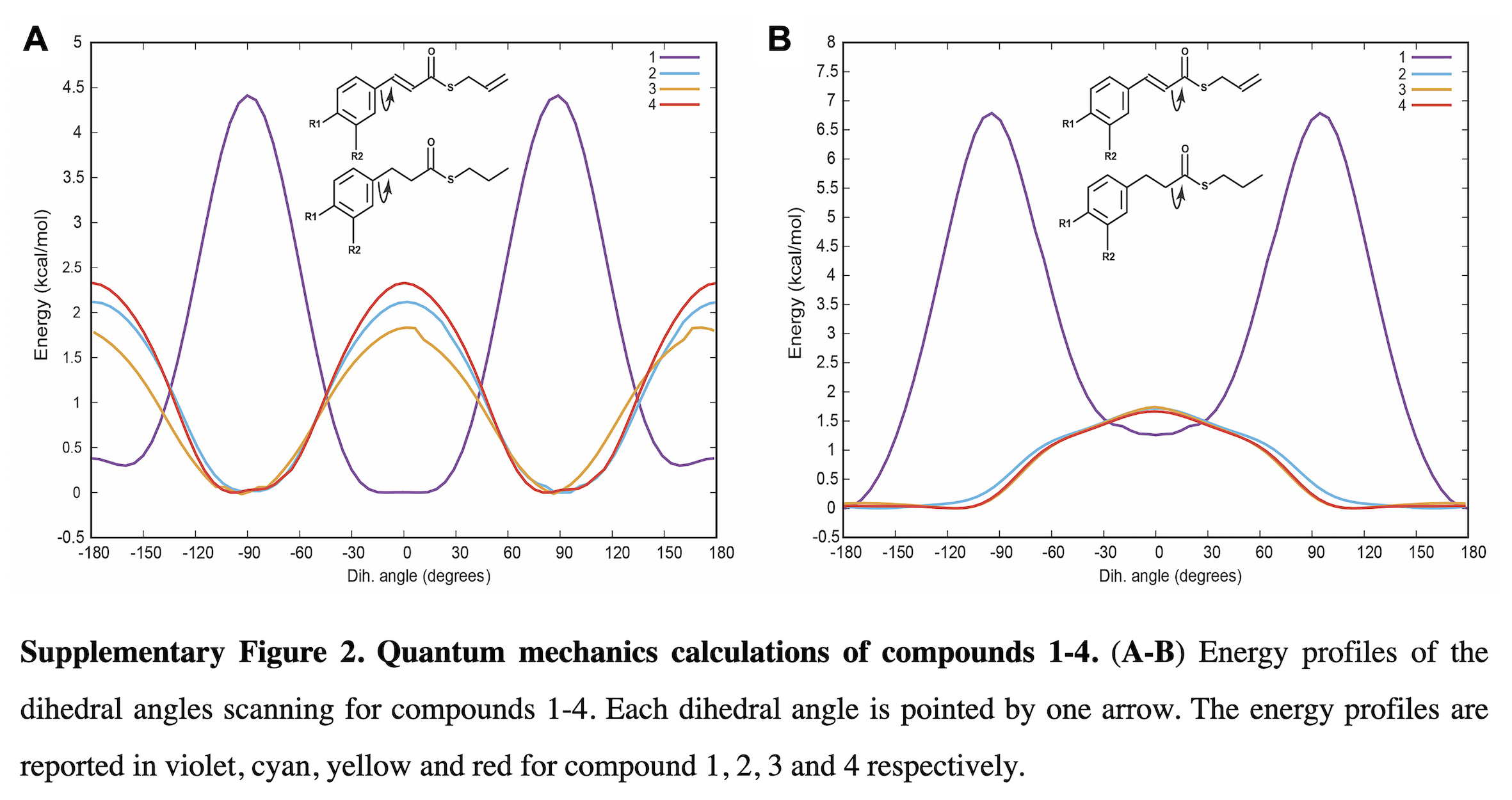

Supplement: Supplementary file 2 [file Image_2.tif]

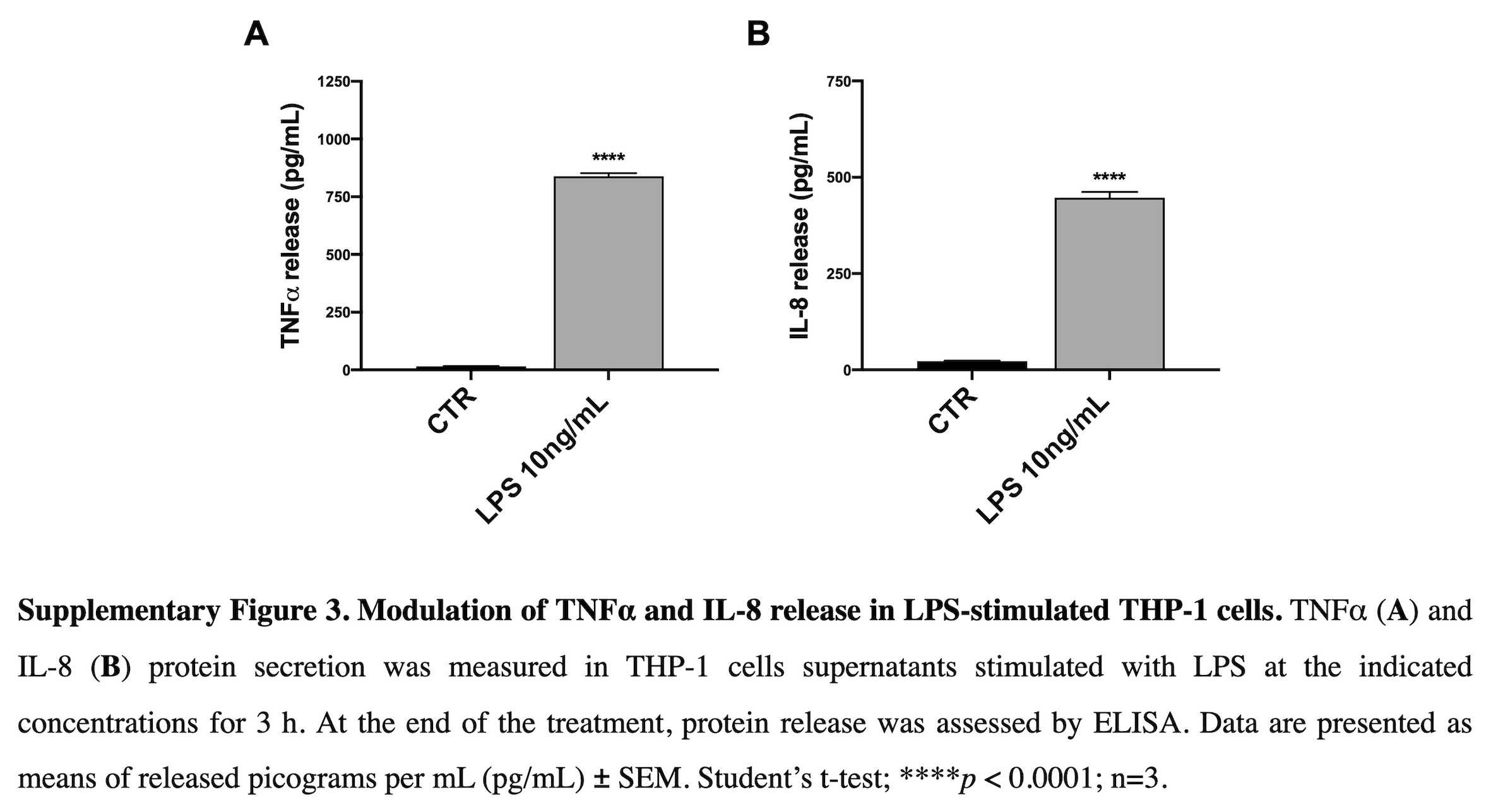

Supplement: Supplementary file 3 [file Image_3.tif]
